# Construction of Environmental Synthetic Microbial Consortia: Based on Engineering and Ecological Principles

**DOI:** 10.3389/fmicb.2022.829717

**Published:** 2022-02-23

**Authors:** Yu Liang, Anzhou Ma, Guoqiang Zhuang

**Affiliations:** ^1^Research Center for Eco-Environmental Sciences, Chinese Academy of Sciences, Beijing, China; ^2^College of Resource and Environment, University of Chinese Academy of Sciences, Beijing, China

**Keywords:** synthetic biology, cross-feeding, traits, spatial structure, microbial consortia

## Abstract

In synthetic biology, engineering principles are applied to system design. The development of synthetic microbial consortia represents the intersection of synthetic biology and microbiology. Synthetic community systems are constructed by co-cultivating two or more microorganisms under certain environmental conditions, with broad applications in many fields including ecological restoration and ecological theory. Synthetic microbial consortia tend to have high biological processing efficiencies, because the division of labor reduces the metabolic burden of individual members. In this review, we focus on the environmental applications of synthetic microbial consortia. Although there are many strategies for the construction of synthetic microbial consortia, we mainly introduce the most widely used construction principles based on cross-feeding. Additionally, we propose methods for constructing synthetic microbial consortia based on traits and spatial structure from the perspective of ecology to provide a basis for future work.

## Introduction

The concept of synthetic biology was developed in the last century. Synthetic biology is an applied discipline in which engineering principles are applied to system design (Kitney and Freemont, 2012). The goal of synthetic biology is to design and manipulate bio-based parts, devices, and systems to create new functions. It can also be used to redesign existing natural biological systems. Synthetic biology has extensive applications in the fields of energy, medicine, and environmental science. An important emerging area of research in synthetic biology is the development of synthetic microbial consortia, which refers to artificial consortia systems constructed by co-cultivating two or more microorganisms under certain environmental conditions (De Roy et al., 2014; Grosskopf and Soyer, 2014). Synthetic microbial consortia have wide applications and represent the intersection of synthetic biology and microbiology.

**FIGURE 1:**
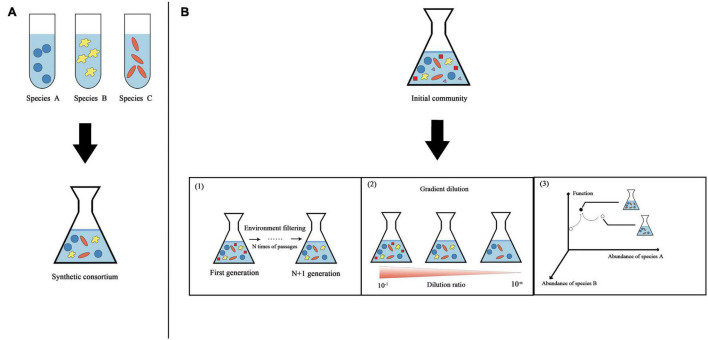
Overview of two classic strategies used to obtain synthetic microbial consortia. **(A)** “Bottom-up” strategy. Different species are assembled according to rules to obtain synthetic consortia with specific functions. **(B)** “Top-down” strategy. Using natural microbial communities, physical and chemical parameters are optimized in a bioreactor to maximize the community function. Three specific methods for obtaining synthetic consortia are shown here based on this strategy. (1) Continued enrichment. A stable synthetic consortium is obtained through ongoing environmental filtering of the original community. (2) Gradient dilution. Gradient dilution of the original community to extinction is performed to obtain a synthetic consortium with relatively few species but functional stability. (3) Directed evolution. Microbial community structure-function graphs are constructed from multiple stable communities with different functions. The community state in the graph is determined by the abundance of different species in the stabilized community (this graph is simplified to show only two dimensions). We first select stable communities with relatively strong functions from the community library and then apply ecological perturbations to generate neighborhood variation; thereby, the communities gradually approach stable states with maximal functions.

Synthetic microbial consortia, like synthetic biology broadly, are based on a closed-loop research method of design-build-test-learn (DBTL) (Lawson et al., 2019) and have the characteristics of engineering. Two classic engineering strategies, “top-down” and “bottom-up,” are used to obtain synthetic microbial consortia (Figure 1). In addition to these two classic strategies, there is also a construction strategy that combines the two strategies.

### Top-Down

Assembly is the main concept in the “top-down” strategy. This strategy refers to the establishment of a stable co-cultivation system for multiple bacterial groups in accordance with certain principles to perform desired functions. Synthetic microbial consortia formed by this strategy have been shown to be more effective than single strains in terms of the synthesis or degradation of organic matter (Zuroff and Curtis, 2012). However, the identification and culture of microorganisms is an important prerequisite for this strategy. Although various techniques, such as in situ culture, high-throughput culture, resuscitation stimulation, and cell sorting, have promoted research on uncultured microorganisms, most microorganisms remain unculturable (Xie et al., 2021). Furthermore, the long-term regulation of community structure is a challenge to this strategy. In long-term cultivation, different microbial taxa compete for the same resources, and one species tends to be dominant.

### Bottom-Up

A selection process is the basis for this strategy. Using natural microbial communities, physical and chemical parameters are optimized in a bioreactor to maximize the community function and finally obtain the minimal active microbial consortia (MAMC) (Puentes-Tellez and Falcao Salles, 2018). They are critical players in a complex ecosystem. MAMC have the same division of labor as “top-down” consortia, yet retain functional redundancy. They are co-evolved from a group of microorganisms under certain culture conditions, and may have better temporal stability than that of “top-down” consortia (Gilmore et al., 2019). However, the acquisition of the MAMC is random, and it is difficult to determine the direction of evolution.

This strategy involves two main methods: continuous enrichment (Gilmore et al., 2019) and serial dilution (Diaz-Garcia et al., 2021). However, these two methods are random and cannot guarantee the ecological balance of the synthetic consortium. Some studies combine directed evolution to propose a method to find stable synthetic consortia based on a top-down strategy. Chang et al. (2021) constructed a multidimensional coordinate system of microbial community structure-function, containing multiple stable communities with different functions. Community status is determined by the abundance of different species in stable communities. A community with strong function is selected from the community library, and communities showing variation are generated by ecological disturbance, gradually approaching a stable community with the maximum function. Types of ecological disturbance include species invasion, species elimination, random small displacement of nutrients, etc. The directed evolution of communities can enable the efficient selection of ecologically stable functional synthetic consortia.

### Multi-Strategy

To address the shortcomings of the “top-down” and “bottom-up” strategies, researchers have recently tried to combine these strategies to design synthetic microbial consortia. We can assemble multiple members into consortia according to the principle of metabolic networks and then achieve enhanced functions by changing environmental variables to regulate the consortia. Controlling the inoculation rate is the most common method for the assembly of multiple consortium members; however, synthetic microbial consortia constructed by this method often deviate rapidly from the initial conditions. A method based on temperature cycling has been proposed (Krieger et al., 2021) to effectively control a synthetic microbial consortia assembled by *Escherichia coli* and *Pseudomonas putida*. Many environmental variables can be altered to adjust the composition of the synthetic microbial consortia, like temperature, and thereby to adjust the metabolic function of the consortia. These methods are still essentially based on ecological mechanisms.

Different strategies have been applied to the construction of synthetic microbial consortia. Table 1 gives examples of the construction of synthetic microbial consortia by different strategies.

**TABLE 1:** Development of synthetic microbial consortia by different construction strategies.

Strategy	Function	Results	Type of microorganism	Refs.
Top-down	Degradation of 1,2,3-trichloropropane (TCP)	The conversion rate of TCP into glycerol was 78%.	*Rhodococcus rhodochrous* NCIMB 13064 and *Agrobacterium radiobacter* AD1	Dvorak et al., 2014
Top-down	Degradation of alkanes	The alkane degradation rate was higher than that of the pure bacteria system and reached 97.41%.	*Acinetobacter* sp. XM-02 and *Pseudomonas* sp.	Chen et al., 2014
Bottom-up	Degradation of lignin	The lignin degradation rate was up to 96.5%.	*Stenotrophomonas maltophilia, Paenibacillus* sp., *Microbacterium* sp., *Chryseobacterium taiwanense*, and *Brevundimonas* sp.	Puentes-Tellez and Falcao Salles, 2018
Bottom-up	Degradation of FTOHs (including 8:2, 6:2, 4:2 FTOHs)	The synthetic microbial consortia converted 20% of 8:2FTOH, 60% of 6:2FTOH, and 70% of 4:2FTOH using n-octane as a co-substrate.	*Pseudomonas butanovora* and *Pseudomonas fluorescens*	Lewis et al., 2016
Bottom-up	Degradation of lignocellulose and chlorophenol	After 9 days, 75% of chlorophenol was degraded; after 12 days, 41.5% of straw was degraded.	*Paenibacillu*s sp. and *Pseudomonas* sp.	Liang et al., 2018
Top-down	Degradation of the herbicide bispyribac sodium (BS)	Maximum BS degradation reached 85.6%.	*Achromobacter xylosoxidan*s (BD1), *Achromobacter pulmonis* (BA2), and *Ochrobactrum intermedium* (BM2)	Ahmad et al., 2018
Multi-Strategy	Strain coexistence experiment	The ratio of the two strains in the synthesis system can be controlled by temperature regulation.	*E. coli* and *Pseudomonas putida*	Krieger et al., 2021

## Application of Synthetic Microbial Consortia in Environmental Science

Synthetic microbial consortia have important significance in many fields, such as bioprocessing (Shong et al., 2012) and fermentation (Wang et al., 2016). But we pay more attention to its application in the environmental field. The value of the synthetic consortium in the environmental field is mainly in two areas: ecological restoration and ecological theory.

### Ecological Restoration

Restoration ecology using synthetic microbial consortia mainly involves two major aims, the degradation of pollutants and the restoration of biodiversity. Pure bacterial systems may show good performance in the laboratory but often face challenges when applied to actual contaminated sites (Liang et al., 2020). Synthetic microbial consortia may show better adaptability and even higher degradation efficiencies than those of pure bacterial systems. For example, *Acinetobacter* sp. XM-02 and *Pseudomonas* sp. have been used to degrade alkane (diesel and crude oil) pollution (Chen et al., 2014). *Acinetobacter* sp. XM-02 can degrade alkane alone. Although *Pseudomonas* sp. cannot degrade alkanes, it can produce surfactants that increase the contact of microorganisms with the oil surface by reducing the surface tension of the medium. The degradation rate of alkane by the co-culture system was 8.06% higher than that of a single strain. Community or species inoculation is one approach to restore ecological diversity. It is mainly used in the restoration of saline-alkaline soil or soils with reduced soil fertility. In a previous study (Anees et al., 2020), the efficacy of phytoremediation of saline-alkaline soil was improved by combining halotolerant and chitinolytic bacteria. Halotolerant bacteria have an antagonistic effect on plant pathogens, and chitinolytic bacteria may help plants escape salt stress.

### Ecological Theory

The assembly of microbial consortia can provide insight into the mechanism underlying microbial community formation and the link between the structure of microbial communities and ecological functions. The phylogenetic limiting similarity hypothesis (PLSH), a classic hypothesis in ecology, suggests that the struggle for existence is stronger between more closely related species. Violle et al. (2011) used bacterivorous ciliated protist species as samples and established a number of synthetic microbial consortia with pairwise interactions in different microcosms to evaluate the relationship between phylogenetic correlation and competitive interactions in ecological communities. The complexity of natural microbial communities may make it difficult to control artificially imposed factors and interpret patterns (Emery and Gross, 2007; De Roy et al., 2014). Synthetic microbial consortia have interspecific interactions similar to natural microbial communities for analyses of associated community changes. Therefore, synthetic microbial consortia have become a powerful tool for ecological theory.

## Construction of Synthetic Microbial Consortia Based on Cross-Feeding

### Definition and Application of Cross-Feeding

**FIGURE 2:**
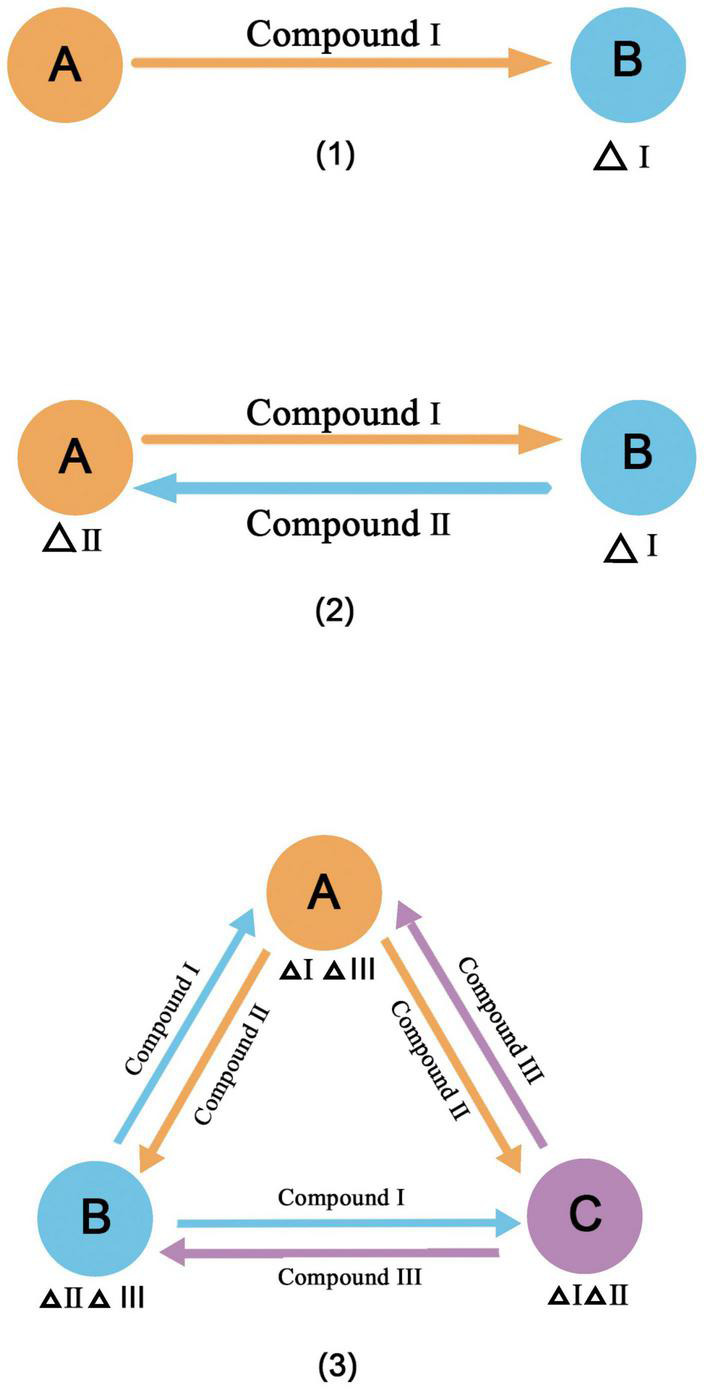
Three forms of cross feeding **(1)** unidirectional **(2)** bidirectional **(3)** multidirectional. Δindicates the lack of materials.

Cross-feeding is a common principle for constructing synthetic microbial consortia. It is a common process in natural communities (Zelezniak et al., 2015) and is considered to contribute to the maintenance of the diversity and stability of natural microbial communities (Klitgord and Segre, 2011; Tsoi et al., 2018). When a metabolite is fully present in the environment, microorganisms may lose some functions due to mutations and form auxotrophs (D’Souza and Kost, 2016). However, neighboring microorganisms that produce the metabolite can become a new source, and they build an interaction network for cross-feeding (Pande et al., 2014). A broad definition of cross-feeding has been proposed (Fritts et al., 2021), in which four requirements must be met. (1) The compounds must be transferred from the producer to the receiver. (2) The transferred compounds must be taken up by the receiver or participate in energy conversion. (3) The adaptability of the producer and the receiver will change due to the acquired compounds. (4) Cross-feeding must involve different species or groups of different genotypes. Cross-feeding compounds vary; the most common are metabolites, followed by extracellular enzymes, siderophores, and so on. In addition, cross-feeding can be unidirectional, bidirectional, and multidirectional (Figure 2), and species or groups vary from two to many. This is a simple method for the classification of cross-feeding, and more specialized classifications have been developed (Smith et al., 2019). The most common example of cross-feeding in nature is the degradation of lignocellulose (Lee et al., 2021). In this process, each member of the microbial community completes a step in the degradation pathway, releasing an intermediate product to be used by the next member, and some members degrade toxic intermediates. There is also cross-feeding of product exchange through microbe-microbe surface interactions, such as the use of electrically conductive pili (epili) for electron transport by community members during methanogenesis (Rotaru et al., 2014). When microorganisms exchange metabolites by cross-feeding and all members can benefit from this exchange, this is referred to as functional specialization or division of labor (Giri et al., 2019). We can build synthetic microbial consortia based on cross-feeding by reasonable microbial division of labor. The community not only retains some of the complexity of natural communities, such as the ability to strengthen ecosystem functions and resist environmental disturbances (Sieuwerts et al., 2008; Spus et al., 2015), but also uses interactions to increase productivity or resource utilization (Bernstein et al., 2012; Jones et al., 2017). Table 2 shows examples of the application of cross-feeding principles to construct synthetic microbial consortia.

**TABLE 2:** Construction of synthetic microbial consortia based on cross-feeding.

Member	Key metabolites	Description of cross-feeding	Refs.
*E. coli* K-12 MG1655	Glucose	*E. coli* A cannot use glucose but can consume by-products produced by *E. coli* B; *E. coli* B can use glucose.	Bernstein et al., 2012
*Dehalococcoides mccartyi* and *Desulfovibrio vulgari*s Hildenborough	CO	*Dehalococcoides mccartyi* produces toxic CO when it degrades chlorinated solvents alone. *Desulfovibrio vulgaris* Hildenborough consumes CO.	Zhuang et al., 2014
*Pseudomonas putida*, *Cellulomonas fimi*, *Yarrowia lipolytica*, and *Methylorubrum extorquens*	Methanol	*Methylorubrum extorquens* consumes toxic methanol produced during lignin degradation.	Lee et al., 2021
*E. coli* and *Rhodopseudomonas palustris*	Carbon and NH_4_^+^	*E. coli* provides carbon to *Rhodopseudomonas palustris* in the form of glucose fermentation products; *Rhodopseudomonas palustris* provides NH_4_^+^ to *E. coli*.	LaSarre et al., 2020
*Variovorax* sp. WDL1, *Delftia acidovorans* WDL34, and *Comamonas testosteroni* WDL7	3,4-DCA	*Variovorax* sp. WDL1 grows with linuron as a carbon and nitrogen source and degrades it into intermediate products. *Delftia acidovorans* WDL34 and *Comamonas testosteroni* WDL7 then degrade these intermediate products.	Dejonghe et al., 2003

A combination of mathematical modeling and biology has been used to design and construct synthetic microbial consortia. Ecological models describing microbial interactions have been developed, such as the generalized Lotka–Volterra (gLV) and consumer-resource (C-R) models (Coyte et al., 2015; Niehaus et al., 2019). However, the parameters are often highly complex and substantial data are required for model fitting. Therefore, a series of optimizations based on these models have been developed to enable the description of more complex communities. Liao et al. (2020) established a coarse-grained metabolic model to simulate unidirectional, bidirectional, and multidirectional cross-feeding and accurately predicting the response to interference. Zelezniak et al. (2015) described a genome-scale modeling method to study the complex cross-feeding of multiple species and substances in the natural environment. In addition to ecological models, metabolic network models are used for the construction and subsequent regulation of synthetic microbial consortia. Flux balance analysis (FBA) is a typical constraint-based metabolic network model (Biggs et al., 2015; O’Brien et al., 2015; Biggs and Papin, 2017). It uses linear programming to determine the metabolic flux distribution of the reaction network based on gene annotations and biochemical reactions in the literature. FBA can optimize the metabolic network and predict gene knockouts expected to produce the best metabolic response (Burgard et al., 2003), providing guidance for the construction of synthetic microbial consortia based on cross-feeding.

Quorum sensing (QS) can also tune some intermediate metabolites for cross-feeding. Quorum sensing is a density-dependent microbial mechanism. When the concentration of a signaling substance reaches a threshold level, some microbial activities are activated and the phenotype changes. QS can regulate the secretion of extracellular digestive enzymes (Allen et al., 2016) to coordinate the cooperation of *Pseudomonas aeruginosa*. Guo et al. (2021) also found that some species can provide expensive metabolites to DSF-secreting species after the addition of exogenous diffusible signal factor (DSF) to the anammox microbial co-culture system.

Cross-feeding increases target yields and improves metabolic activity compared to those for single species. Cross-feeding combined with metabolic engineering can reconstitute heterologous metabolic pathways in different species and use a variety of tools and strategies to maximize metabolic flux of target products (Park et al., 2019). Metabolic engineering can optimize the operating efficiency of the synthetic consortium, and the combination of rapid product detection technology, metabolomics (Zhalnina et al., 2018a), isotope tracing (Gebreselassie and Antoniewicz, 2015) and other testing technologies can shorten the “design-build-test-learn (DBTL)” cycle time. Combining computational techniques with metabolic engineering can also shorten the period of the DBTL cycle. Nakazawa et al. (2021) developed a specific vocabulary model. The model searches relevant literature in the fields of synthetic biology and metabolic engineering to suggest next steps for genetic modification based on the relevance of the genetic modification history. This model can also be extended to the optimization of synthetic consortium DBTL loops.

Additionally, metabolic engineering can exploit the strengths and interactions of different species to improve productivity. Zhou et al. (2015) used *E. coli* and *Saccharomyces cerevisiae* to produce paclitaxel precursors, using the rapid growth of *E. coli* and the advanced protein expression mechanism of *S. cerevisiae* to improve the production efficiency of paclitaxel. Cross-feeding and co-cultivation can also improve the metabolic activity. Many bacteria enter a viable but not culturable (VBNC) state under adverse environmental conditions. In this state, the metabolic activity of microorganisms will be reduced and the function of the community will be affected (Wang et al., 2020). But co-cultivation and cross-feeding can convert some VBNC bacteria into a culturable state for them to function (Senoh et al., 2010).

### Disadvantages of Synthetic Microbial Consortium Construction Based on Cross-Feeding

Although cross-feeding is commonly used for the construction of synthetic microbial consortia, this strategy has some disadvantages. These disadvantages are mainly related to the maintenance of the stability of the consortia.

First, although it is generally believed that cross-feeding is a cooperative interspecific relationship, there is still competition between species. Studies have pointed out that there are almost no examples of real interspecific cooperative relationships (De Mazancourt et al., 2005; Oliveira et al., 2014). As a typical example, *Prochlorococcus* provides a carbon source for the marine bacterium SAR11, but the species compete for sulfur resources (Becker et al., 2018).

Second, although cross-feeding is theorized to show multiple stable states (Vet et al., 2018), ecological stability does not indicate evolutionary-genetic stability (Miller and Klausmeier, 2017). Co-cultivation of species with cross-feeding relationships may lead to functional enhancement. Hillesland et al. (2014) cultured *Desulfovibrio vulgaris* with *Methanococcus maripaludis* and found that over the first 300 generations, the co-culture evolved higher compound yields and faster growth rates. However, co-cultivation can also lead to the breakdown of the cross-feeding system. The consortia based on amino acid cross-feeding (Wintermute and Silver, 2010) may be stable in the short term. However, since the output of amino acids is not fixed, when this output is insufficient during environmental selection (Kerner et al., 2012), the steady state will be destroyed. Mutations can also disrupt the stability of synthetic microbial consortia. Cheaters can easily arise by genetic mutations that produce functional gene deletions (Pande et al., 2014). Cheaters benefit from mutually beneficial and symbiotic interactions but cannot contribute to the system, ultimately leading to the collapse of the cross-feeding system. When cheaters have an advantage in the consumption of intermediate metabolites in cross-feeding, they may lead to the extinction of some species, including cross-feeding species (Sun et al., 2019).

The details of intermediate metabolites affect the function of the consortia. The production of intermediate metabolites may determine whether the metabolites are beneficial. LaSarre et al. (2017) used *E. coli* and photoheterotrophic *Arabidopsis* to construct a synthetic system based on cross-feeding. In this system, *Arabidopsis* produces NH_4_^+^ as an ammonia source and *E. coli* releases organic acids to provide carbon sources for *Arabidopsis*. However, when *Arabidopsis thaliana* secretes high levels of NH_4_^+^, it will stimulate *E. coli* to produce more toxic organic acids. This phenomenon is called dose-dependent toxicity. This phenomenon also exists in the microbial co-culture system. LaSarre et al. (2017) constructed an anaerobic co-culture system of fermentative *E. coli* and *Rhodopseudomonas palustris*, and the exchanged metabolites were carbon (organic acids) and nitrogen (ammonium). When *R. palustris* increased NH_4_^+^ production, *E. coli* organic acid production also increased. This results in acidification of the culture system, leading to an imbalance in species ratios and reduced carbon conversion efficiency. In addition, the feed the faster grower hypothesis (FEG) (Hammarlund et al., 2019) also suggests that when intermediate metabolites are added to a bidirectional cross-feeding system, the coexistence of consortium members depends on relative growth rates. If the faster-growing species loses its dependence on another species by utilizing alternative sources, the slow-growing species will die. In addition, intermediate metabolites may become toxic substances due to environmental factors, such as pH (Daims et al., 2016). In addition to intermediate metabolites, some inhibitors of the fermentation process also inhibit the efficiency of the synthetic consortium. The production of lactic acid from lignocellulose is a common function of synthetic consortia (Shahab et al., 2018). However, the process is challenging due to inhibitory by-products. They include phenolic compounds from lignin degradation, furan from sugar degradation, fatty acids, inorganic ions, and bioalcohols (Zhang et al., 2016). These compounds inhibit fermentation substantially by affecting cell growth and enzymatic activity, hindering lignocellulose utilization and efficient fermentation.

External interference also has an impact on synthetic microbial consortia based on cross-feeding. For example, due to the short-board effect, in a multidirectional cross-feeding system, antibiotic resistance of the consortia often depends on the most intolerant member (Adamowicz et al., 2018). It is also possible that when a key member of the consortium is disturbed and dies due to external interference, a mutually beneficial symbiosis will turn into a competitive relationship, leading to the collapse of the system (Hammarlund et al., 2021).

The concept of cross-feeding spans multiple disciplines, such as ecology and metabolomics. It is not easy to construct and maintain synthetic microbial consortia based on cross-feeding, and this approach requires insights from various perspectives.

## Synthetic Microbial Consortia Construction—Inspiration From Community Ecology

A community is a complex of animals, plants, and microorganisms that inhabit the same area. Many ecological processes are often not completed by a species but by communities of multiple species acting together. Therefore, community ecology research is of great significance.

In studies of animal and plant communities, many theoretical models have been developed to explain ecological phenomena (Dt, 1982; Jetschke, 2002). However, due to complex interspecific relationships, high mutation rates, high rates of horizontal gene transfer (HGT), and other factors, microbial communities often show distinct characteristics from those of animal and plant communities (Head and Editor, 2007), making the application of theoretical models difficult. In particular, several key issues are as follows. (1) Many microorganisms cannot be cultivated in the laboratory, limiting our ability to test theoretical models. (2) Environmental conditions are important determinants of microbial communities (Wilpiszeski et al., 2020; Chen et al., 2022). (3) In animal and plant communities, population dynamics modeling is used to study relationships within the community matrix; however, when there are a large number of populations in the community, some model parameters are difficult to determine (Novack-Gottshall, 2000). This issue was also mentioned in our discussion of the construction of synthetic microbial consortia based on cross-feeding. Advances in molecular biology technologies have provided an opportunity to apply theories and methods developed for animal and plant communities to microbial communities, especially in microbial biogeography (Martiny et al., 2006). However, there are still limitations, including complex data processing (Raes and Bork, 2008), complex genotype–phenotype relationships (Temperton and Giovannoni, 2012), and complex interactions.

Treating the synthetic microbial consortia as a “community,” the phenotype of a component species may be different from that of the species cultivated separately. Additionally, unlike a single species, synthetic microbial consortia retain some of the complexity of natural communities and therefore can perform more functions than is possible by a species (Spus et al., 2015; Jones et al., 2017). The cross-feeding strategy is still the most common approach for constructing synthetic microbial consortia. As mentioned above, this strategy still has many disadvantages, mainly the difficulty in maintaining the stability of the synthetic consortium.

The synthetic consortium is similar to the natural community, and its long-term functional maintenance depends on its stability, i.e., the ability of the community to maintain or return to its original state after disturbance (Pimm, 1984). Stability itself is a multi-dimensional concept. Common indicators for stability include resistance stability and resilience stability. Resistance stability is the ability of a community to resist external disturbances and maintain its original structure and function. Resilience stability is the ability of a community to return to its original state after being damaged by external disturbance factors. Community stability is associated with species diversity, which also presents challenges for synthetic consortia because synthetic consortia are often the result of the simplification of species in natural communities. The reduction of species diversity leads to the loss of functional redundancy, which is the key to community functional stability (Shade et al., 2012). Additionally, different from natural communities, the functional stability of synthetic consortia is affected by several other factors. (1) Synthetic consortia tend to be cultured in larger numbers and for longer periods of time in industrial applications, leading to the loss of specific phenotypes (Rugbjerg et al., 2018). (2) Changes in environmental conditions during the cultivation process can reduce stability, particularly the accumulation of toxic products and changes in environmental pH. (3) Spatiotemporal heterogeneity of the availability in resources (such as nutrients and oxygen) can affect overall metabolism (Muller et al., 2010).

Because the construction method based on cross-feeding cannot well consider the stability of synthetic consortia, we refer to the emergence of many new theories and methods in community ecology in recent years, which provides new directions for the construction of synthetic microbial consortia.

## Construction of Synthetic Microbial Consortia Based on Traits

### Concept of Traits

Traits refer to the (phenotypic) characteristics that can be observed in individuals, including biochemical characteristics, cell morphology or dynamic processes, anatomical structures, organ functions, or behavioral characteristics (Violle et al., 2007). Traits can be classified according to their complexity. For example, some traits are controlled by a single gene and can be regarded as simple traits; other traits are controlled by multiple genes and may also be affected by the environment and these are referred to as complex traits (Frankham, 1996). Traits can also be classified into continuous traits, such as salt tolerance at different temperatures, and discrete traits, such as the ability of microorganisms to fix nitrogen or carbon dioxide. Traits can also be divided into response traits and effect traits (Webb et al., 2010). Response traits are related to the response of organisms to environmental changes (such as drought tolerance); effect traits refer to the impact of organisms on ecosystem processes (such as photosynthetic capacity). Additionally, functional traits are any morphological and physiological characteristics of microorganisms in a specific environment, such as size, reproduction time, or response to a certain antibiotic (McGill et al., 2006).

Trait-based methods are increasingly used in ecology to study ecological processes and community assemblies. They have been applied to studies of animals (Frimpong and Angermeier, 2010), higher plants (Van Bodegom et al., 2012), and plankton (Litchman and Klausmeier, 2008). Taking plants as an example, a trait-based method has been proposed (Van Bodegom et al., 2012) to describe global vegetation functions able to predict the continuous response of plant communities to multiple environmental variables. This method resolves the shortcomings of using plant function types (PFTs) to describe ecosystem functions. Compared with individual-based models commonly used in ecology, the trait-based model can better capture correlations between community functions and environmental changes (Zakharova et al., 2019). Kruk et al. (2021) used trait-based modeling to analyze how trait-related differences in species fitness affect phytoplankton species invasion.

Microbial ecologists have recently used trait-based models for plants or plankton for reference in microbial ecology research, providing a new framework for studying the geographic distribution, function, and trends of microbial communities (Wallenstein and Hall, 2011). Fungi were an initial research object. Chaudhary et al. (2020) collected morphological and functional traits of arbuscular mycorrhizal fungal spores to study the influence of random processes (diffusion) on fungal community structure. Studies have found that arbuscular mycorrhizal (AM) fungal spores in the air exhibit traits that are more conducive to airborne transmission (such as a smaller average spore size). Guittar et al. (2019) used a trait-based model to study the succession of the infant intestinal flora and found that the community composition was basically stable after one year. Predicted values for most traits become more accurate over time, indicating that succession is at least partially functionally deterministic. In studies of environmental microorganisms, trait-based methods have also been developed to predict primary productivity (Follows et al., 2007), metabolism (Hall et al., 2008), and litter decomposition (Allison, 2012). For example, enzyme kinetics and physiological traits have been used to represent ammonia-oxidizing bacteria (AOB), ammonia-oxidizing archaea (AOA), and nitrite-oxidizing bacteria (NOB). Trait-based modeling has been used to successfully predict various functional changes along environmental gradients (Bouskill et al., 2012).

Trait-based methods are of great significance in microbial ecology. This approach makes up for the shortcomings of analyses based on taxonomy, functional genes, or protein sequences, and more effectively associates microbial communities with ecosystem functions. However, microbial ecology based on traits is still in its infancy (Krause et al., 2014) for two primary reasons. First, it is difficult to define which traits are important. For many microorganisms, the key traits that determine environmental adaptability are still unclear. Second, it is difficult to quantify and characterize microbial traits. The metabolic diversity of bacteria, genomic plasticity, and trade-offs between traits make the simple division of environmental microorganisms into functional groups impossible. Furthermore, to study traits at the community level, the definition of community-weighted trait means (Violle et al., 2007) alone is far from sufficient because this may conceal local functional diversity.

New technologies have facilitated the measurement and quantification of microbial traits. For example, microbial culture technologies have been developed. Microfluidic technology (Sanati Nezhad, 2014) expands the range of microorganisms that can be cultivated, allowing us to obtain more information about the size, metabolic rate, and nutrient utilization of microorganisms. Isotope tracing methods include stable isotope tracing (Casey et al., 2007) and nano-SIMS (Behrens et al., 2012) and can be used to study the utilization of substrates by microorganisms at different scales. Raman spectroscopy (Huang et al., 2007) can reveal the chemical composition of cells. The development of sequencing technologies and omics methods has also been useful for trait-based research. Metabolomics (Li et al., 2021) can characterize the ability of microorganisms to utilize different substrates. Metagenomics (Wrighton et al., 2012) has improved our understanding of uncultivated microorganisms. Single-cell sequencing (Martinez-Garcia et al., 2012) can be used to study uncultured microorganisms and to accurately distinguish the genotype of each cell. However, it is important to account for the influence of the environment on phenotypes when using omics and sequencing technologies. Increasing studies have evaluated associations between microbial genotypes and phenotypes (Green et al., 2008; Martiny et al., 2015). In the near future, it may be possible to determine traits based on genotype data. A future goal is to establish a standardized trait database, like that available for plants, for easy access to information.

### Application of Trait-Based Methods to Synthetic Microbial Consortia

The construction of synthetic microbial consortia involves many principles of microbial ecology. Regardless of whether we adopt a “top-down” or “bottom-up” strategy, it is necessary to determine the number of species that should be retained/assembled to maximize the function of the synthetic microbial consortia. Krause et al. (2014) concluded that diversity and functionality mainly involve three major modes of action: complementary effects, selection effects, and promotion effects (inhibitory effects). These mechanisms provide a basis for manipulating diversity, thereby altering community functionality. We also need to consider community stability. Functional redundancy may be the key to community stability (Louca et al., 2018). The construction of synthetic groups based on cross-feeding is typically only considered from a functional perspective and does not consider functional redundancy and stability. Triado-Margarit et al. (2019) used a trait-based model to determine an environmental threshold, above which community traits tend to be stable, there is indicating that there is functional redundancy. This case uses the definition of traits to better understand the end point (steady state) of community assembly.

Research in microbial ecology is traditionally based on taxonomy, with the classification of groups based on the current species definition (Schleifer, 2009). With the development of molecular biotechnology, methods based on functional genes or protein sequences have been applied to microbial ecological research. However, such methods are often susceptible to the plasticity of traits, leading to differences in genotype and phenotype (Coutinho et al., 2016). Salles et al. (2009) found that the complementary effects of traits relative to taxa or gene or protein sequences can better predict denitrification performance. This indicates that trait-based methods may better describe or predict the functions of the community than traditional taxonomy-based methods.

**FIGURE 3:**
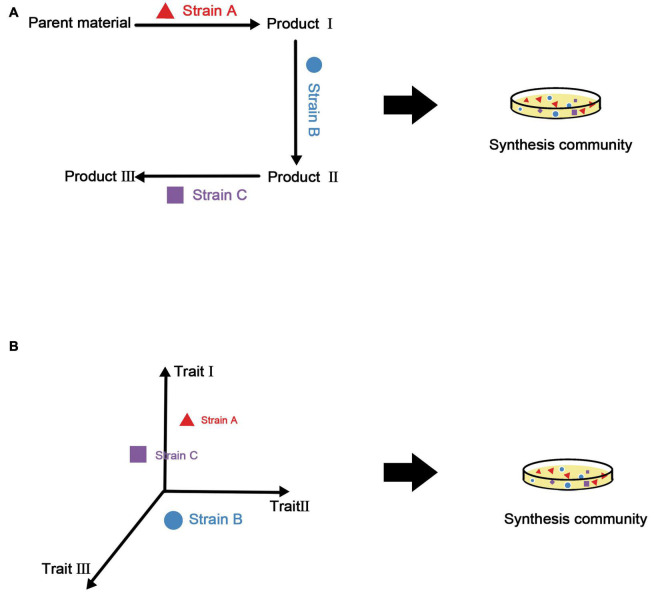
Construction methods for synthetic microbial consortia based on metabolic associations and traits. **(A)** Using metabolic associations, metabolites of various strains are utilized to build a complete metabolic network. Although the functionality of the synthetic microbial consortia may be improved under this method, it is difficult to evaluate the stability of the consortia. **(B)** The trait-based method uses a multi-dimensional model from the perspective of ecological niches to obtain synthetic microbial consortia with good stability and functionality.

Combining the above two points, our strategy for the construction of synthetic microbial consortia based on traits is to select an appropriate combination of features according to the final functional requirements and manipulate diversity to complete the assembly of the consortia. There is an example of this approach in plants (Wanapaisan et al., 2018), in which a model was built based on three traits (i.e., thick bark, dense wood, and a moderate leaf nitrogen concentration) and post-disaster restoration of mountain forests in western North America was carried out by controlling the number of species and introducing specific new species. It is even possible to establish a species library and select taxa with appropriate traits from this library according to the function of the synthetic microbial consortia. Figure 3 summarizes the process of constructing synthetic microbial consortia based on trait models.

Not only for the design and construction phase of synthetic microbial consortia but also for the regulation of synthetic microbial consortia, trait-based models are a good tool. Trait-based methods can use the functional traits of a community in a specific environment as a basis to predict the community’s response to specific environmental changes. Le Roux et al. (2016) used a trait-based model to classify soil NOB into several groups determined by physiological traits, and predicted that NOB will affect three climatic factors (CO_2_ increase, precipitation, and nitrogen addition). Using trait-based models, we can rationally regulate synthetic microbial consortia by adjusting environmental factors. Although trait-based models cover multiple dimensions, there are some current techniques improve the construction of a trait-based synthetic consortium, such as machine learning (Mallick et al., 2019), metabolomics (Zhalnina et al., 2018a) and laboratory ecosystems (Zhalnina et al., 2018b). Mallick et al. (2019) used machine learning to predict metabolites that might not be observed in microbial communities. Metabolomic characteristics of microbial communities are inferred from metagenomes based on the MelonnPan computational framework, successfully predicting more than 50% of metabolites at the community level. Based on computational models, we can better combine a set of optimal microbial traits and infer the metabolites of synthetic consortia.

## Constructing Synthetic Microbial Consortia Based on Spatial Structure

Species composition and structure are two basic characteristics of a community. When understanding microbial communities, we often focus on the community composition, such as species diversity and interactions. In recent years, synthetic microbial consortia have been constructed based on interactions between species (Yurtsev et al., 2016; Li et al., 2017; Ren et al., 2018). However, increasing studies have shown that spatial structure is equally important for the function of microbial communities (Kim et al., 2008).

### Concept of Spatial Structure

**FIGURE 4:**
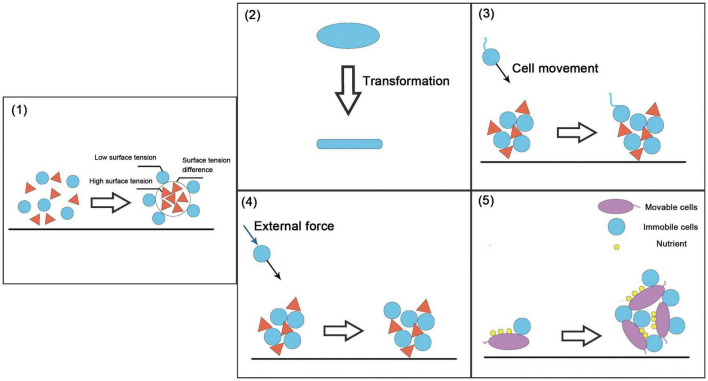
Examples of spatial structure-based construction strategies. **(1)** Self-organized construction strategy (i.e., the differential adhesion hypothesis), in which the difference in surface tension and interfacial tension between moving cells produces selective adhesion. **(2)** Changes in cell shape. **(3)** Movable cells produce specific movements under the action of environmental information. **(4)** Immovable cells move or diffuse with the help of external environmental forces such as liquid flow. **(5)** Immovable cells move via movable cells.

The spatial structure of the community refers to the distribution of the population in the community and the spatial arrangement of its attributes (Ledo et al., 2014). It reflects the interactions among individuals in horizontal space and is a universal characteristic of the community. The spatial structure is the result of the combined effects of population biological characteristics, intra-specific and inter-specific interactions, and environmental conditions (Druckenbrod et al., 2005). There are some key strategies for constructing community spatial structure: the self-assembly of microorganisms, i.e., the “differential adhesion hypothesis” (Steinberg, 1975, 2007) and construction strategies based on cell movement, cell morphological changes, etc. (Figure 4).

Spatial stratification mainly includes two categories in natural communities: resistance to stress and changes in interactions. The spatial structure constructed by multiple species is often stronger than that of a species in these two aspects. For example, compared with the species, the seawater biofilm community is more resistant to antibiotics (Burmolle et al., 2006). In the soil environment, the spatial structure affects the interaction between microorganisms, which in turn affects many phenomena, such as nitrification (Ebrahimi and Or, 2016). It should be noted that the spatial structure can also change metabolite exchange into cross-feeding (Liu et al., 2017), allowing us to simultaneously incorporate cross-feeding approaches to better construct synthetic microbial consortia. The spatial structure of the community helps us understand the structure and function of the community and dynamic population processes and reveals the mechanism underlying the maintenance of species diversity in the natural community. In higher plant and animal ecology, spatial structure is widely used in ecosystem restoration and maintenance.

In the laboratory, microbial systems that can be cultivated are very limited. Many microorganisms cannot produce stable communities when mixed well. This can be explained by unbalanced competition between species for nutrients (Kim et al., 2008). This further demonstrates that the functionality of the microbial community is not only related to diversity but also to the spatial structure of the community (Liu et al., 2016; Pandin et al., 2017).

Compared with higher plant communities, microbial communities are more complex and difficult to observe, with no obvious vertical and horizontal structure. Another key issue in spatial ecology is the scale of analysis. The scale is either too small to characterize the complete community structure or too large to find local characteristics. Technological advances have provided additional means to study the micro-scale spatial structure of microorganisms in the laboratory. Microfluidic technology is an emerging method to study the micro-scale spatial structure of microorganisms. Microfluidic technology can accurately control environmental parameters within a small range and, combined with microscopic imaging technology, can be used to observe biological processes in real time (Sanati Nezhad, 2014). Massalha et al. (2017) used microfluidic-based technology to study the chemotactic behavior of bacteria relative to plant roots. Combined labeling and spectral imaging fluorescence in situ hybridization (CIASI-FISH) can simultaneously identify and locate different microorganisms and further determine the distribution of a certain species. This method extends the observation scale to the community (Wilbert et al., 2020).

### Application of Spatial Structure in Synthetic Microbial Consortia

The micro-scale spatial structure in the natural environment is considered very important for microbial ecology (Treves et al., 2003; Leibold et al., 2004). In the natural environment, bacteria are affected by a series of physical, chemical, and biological factors on the microscale. They ultimately affect some traits on the community scale by affecting the diffusion of bacteria and nutrients (Konig et al., 2020). Although artificial environments created in the laboratory are not as complex as the natural environment, spatial structure is still an important factor. Synthetic microbial consortia constructed by a “bottom-up” strategy can be assembled with different metabolic divisions of bacteria to build a complete metabolic network with certain functions. However, in the actual process of constructing synthetic microbial consortia, the consortia may not perform the ideal function. Because the consortia are affected by species competition, the uniformity of nutrients, and the transmission of signaling molecules and other factors, they cannot grow in a well-mixed condition. Vallespir Lowery and Ursell (2019) used an improved Lotka–Volterra model in a two-dimensional environment to prove that although two microorganisms cannot coexist under homogenized conditions, they can coexist in a structured environment. Kim et al. (2008) constructed synthetic microbial consortia composed of three kinds of bacteria with nutritional interactions; they found that stability is low when the mixture is good but observed stable coexistence after separation and cultivation using a microfluidic device. This indicates that the micro-scale spatial structure is necessary for the coexistence of species in synthetic microbial consortia. This study also divides the microbial consortia into three levels. The first-level consortium is unstable when it loses its metabolic coupling, that is, it is most stable under uniformly mixed conditions. The second-level consortium is unstable when well mixed, but when the species separate spatially, the consortium gradually becomes stable until the maximum separation distance is reached. The third-level consortium is unstable, irrespective of spatial distance. To build stable synthetic microbial consortium, it may be necessary to determine the spatial distance threshold to maintains the stable coexistence of consortium members.

There are many ways to control the spatial structure of natural communities, and these methods can provide a reference for the control of the spatial structure of synthetic microbial consortia. First, the spatial structure is often generated based on some kind of information in the environment. The most common is the concentrations of metabolites. Guo et al. (2018) found that cells maintain a desired spatial structure based on the local concentration of metabolites. Exopolysaccharide (EPS) promotes aggregation in the biofilm, and changing the EPS concentration can directly trigger a change in the biofilm spatial structure (Ziemba et al., 2016). In addition to directly altering community spatial structure by changing EPS concentrations, chemotaxis is equally important for spatial structure. Chemotaxis is a motor behavioral response of bacteria driven by changes in the concentration of chemical substances in the environment (Berg, 1975). Chemotaxis plays an important role in both rhizosphere microbial colonization and pollutant degradation. Moisture is a common chemotaxis-based regulator. *Azospirillum brasilense* and *Pseudomonas fluorescens* migrate to roots at a faster rate with increasing soil water contents by chemotaxis (Bashan, 1986). The O_2_ concentration is also a common regulatory factor. Koza et al. (2011) found that wild *Pseudomonas fluorescens* SBW25 can construct an O_2_ gradient and generate spatial stratification according to different O_2_ concentrations. For some multi-species populations based on cross-feeding, the spatial organization is driven by metabolite exchange. Therefore, changing the concentration of intermediate metabolites may change the original community structure. Using an ecological approach to change specific species in a community can also change the spatial structure. In a typical example, spraying *Bacillus subtilis* can change the spatial organization of the *Aspergillus flavus* biofilm on citrus leaves (Huang et al., 2012). In a four-component population of *Stenotrophomonas rhizophila*, *Xanthomonas retroflexus*, *Microbacterium oxydans*, and *Paenibacillus amylolyticus*, the least abundant *M. oxydans* is the key species influencing the spatial structure of the community (Liu et al., 2017). QS is also associated with various processes, such as EPS production and biofilm formation, and can also be used to modulate the spatial structure of microorganisms (Zhang et al., 2012; Ma et al., 2018). We can promote the aggregation of specific microorganisms by rationally regulating QS signaling substances. N-Butyryl-L-homoserine lactone (C4-HSL) has been detected in syntrophic *Geobacter* communities, which can promote aggregate formation among different species (Wei et al., 2017). Bordeleau et al. (2015) found that microbial type IV pili gene transcription is enhanced by the signaling molecule cyclic diguanosine monophosphate (c-di-GMP), which promotes *Clostridium difficile* cell aggregation.

**FIGURE 5:**
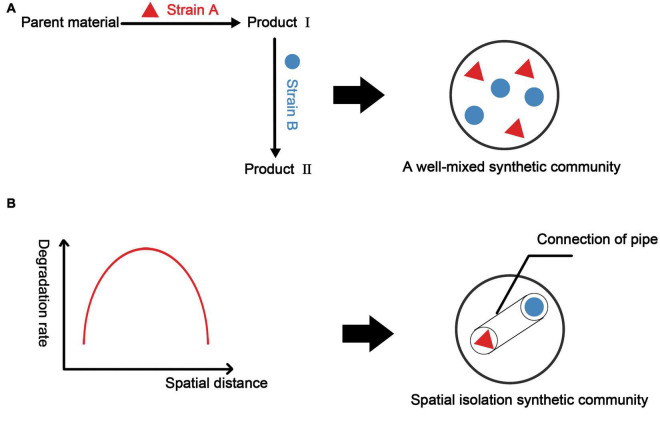
Construction methods for synthetic microbial consortia based on metabolic associations and spatial structure. **(A)** Considering only the metabolic network to construct synthetic microbial consortia, we often obtain well-mixed synthetic microbial consortia. Here, we take two strains as examples. **(B)** Considering the spatial structure, it is assumed that there is a function relating consortium performance to the spatial distance between two strains (in fact, it may be a more complicated spatial model). Based on this function (model), we select the spatial distance with the best consortia performance and construct a vector that can ensure the metabolic communication between the strains by controlling the spatial distance between the two strains.

Some physical methods can be used to directly construct the ideal spatial structure of a biological community. Microfluidics is commonly used to control the spatial structure of microorganisms. Alnahhas et al. used a microfluidic device to control the temporal dynamics of the spatial distribution of two different *E. coli* strains. This study used two cell capture areas of different sizes to control the dynamic ratio of the two strains (Alnahhas et al., 2019). Optical tweezers can capture cells at the single-cell level for precise three-dimensional control (Dholakia and Čižmár, 2011). Miccio et al. controlled the orientation of E. coli by light-induced dielectrophoresis (Miccio et al., 2016). 3D printing technology provides the possibility of artificially constructing the spatial structure of a community, which is often not possible by traditional bioreactors (Rupal et al., 2019). This technology has been applied to mammalian cells. Grix et al. (2018) used 3D printing technology to construct a sinusoidal liver lobule model. The model shows higher protein expression than that in traditional monolayer culture. In environmental sciences, 3D printing technology can be used to build more efficient bioreactors. Elliott et al. (2017) designed a moving bed biofilm reactor (MBBR) with a high specific surface area using 3D printing technology combined with mathematical modeling. However, research on 3D printing technology in environmental sciences has focused on improving the physical and chemical properties of the reactor and has not considered factors such as metabolic coupling. When constructing synthetic microbial consortia, a feasible approach may be modeling and designing the spatial structure of a two-bacteria or even a multi-bacterial combination system and using 3D printing to make a suitable carrier to obtain synthetic microbial consortia or bioreactor with a three-dimensional structure (Figure 5).

## Conclusion and Future Perspectives

The concept of synthetic biology has expanded. It is no longer limited to assembling DNA or organelles. Strains can be used as components to assemble and ultimately build functional consortia. The effective synthetic microbial consortia may show improved survival rates and functions in the natural environment than those of a species. However, the assembly process and underlying principle are more complicated because synthetic microbial consortia are constructed based on laws governing natural communities. It is necessary to consider many factors, including metabolic networks, spatial structure, and stability. In addition to engineering principles, we may also draw inspiration from ecology and apply these insights to the construction of synthetic microbial consortia.

## Author Contributions

YL prepared the draft of manuscript. AM and GZ edited the manuscript. All authors contributed to the article and approved the submitted version.

## Conflict of Interest

The authors declare that the research was conducted in the absence of any commercial or financial relationships that could be construed as a potential conflict of interest.

## Publisher’s Note

All claims expressed in this article are solely those of the authors and do not necessarily represent those of their affiliated organizations, or those of the publisher, the editors and the reviewers. Any product that may be evaluated in this article, or claim that may be made by its manufacturer, is not guaranteed or endorsed by the publisher.
